# Supplementary material for: A Rare and Easily Overlooked Case of Bilateral Traumatic Testicular Dislocation and an Alternative Viewpoint on Delayed Management
Source: Medicina (Kaunas). 2023 May 6;59(5):892. doi: 10.3390/medicina59050892 (PMC10223698; doi:10.3390/medicina59050892)
Supplement: Supplementary file 1 [file medicina-59-00892-s001.zip › Supplementary.pdf]

Table S1: Histological findings of testes in nine cases

Case	Authors	Age	Mechanism	Location	Operation timing	Complications	Pathologic findings	Follow-up semen analysis	Follow-up biopsy
1	Goulding	22	Motorcycle accident	Right inguinal	2 months later	Nil	Absent nuclei of seminiferous tubules, no active spermatogenesis	Nil	Nil
2	Pollen	22	Motorcycle accident	Bilateral inguinal	8 weeks later	Bilateral 180° rotation	No active spermatogenesis	Normal results 3 months later	Nil
3	Nakarajan	25	Motorcycle accident	Bilateral inguinal	6 weeks later	Nil	Diffuse atrophy of the seminiferous tubules, absent sperm formation	Nil	Nil
4	Koga	17	Motorcycle accident	Right inguinal	2 days later	Nil	Normal findings	Nil	Nil
5	Lee	23	Motorcycle accident	Right inguinal	3 months later	Intraperitoneal rupture of the bladder	Hypospermatogenesis with increased Sertoli cells	Oligoasthenospermia 1 month later	Nil

6	Hayami	17	Car collision	Right inguinal	4 months later	180° rotation	Severe impairment in spermatogenesis	Asthenospermia 6 months later	Spermatogenesis slightly improved 8 months later
7	Yagi	25	Fishing boat accident	Left thigh	3 weeks later	180° rotation with slightly torsion	Arrest of spermatogenesis	Nil	Nil
8	Yoshimura	30	Motorcycle accident	Bilateral inguinal	13 years later	Atrophic, bilateral 180° rotation	Severe hypospermatogenesis; Semen azoospermia	Oligospermia improved after 10 months; partner became pregnant	Nil
9	Sakamoto	33	Motorcycle accident	Bilateral inguinal	15 years later	Atrophic, bilateral 180° rotation	Maturation arrest at the primary spermatocyte; Semen azoospermia	Normal results 10 months later; spontaneous pregnancy later	Nil
